# Supplementary material for: Direct presentation of inflammation-associated self-antigens by thymic innate-like T cells induces elimination of autoreactive CD8+ thymocytes
Source: Nat Immunol. 2024 Jul 11;25(8):1367–82. doi: 10.1038/s41590-024-01899-6 (PMC11291280; doi:10.1038/s41590-024-01899-6)
Supplement: Supplementary file 1 — Supplementary Figs. 1–4 and Notes. [file 41590_2024_1899_MOESM1_ESM.pdf]

nature immunology

Supplementary information

https://doi.org/10.1038/s41590-024-01899-6

Direct presentation of inflammation-associated self-antigens by thymic innate-like T cells induces elimination of autoreactive $CD8^+$ thymocytes

In the format provided by the authors and unedited

Supplementary note 1. Contribution of negative selection and lineage diversion to the inactivation of CD8 T cells recognizing an inflammation-associated self-antigen

The overall decrease in total peripheral Jedi cells (Fig. 3e) and the increased cell death of Jedi-TCRαβ DP thymocytes in chimeras with IL4-GFP donors and/or recipients (Fig. 3c,e) suggested that clonal deletion is an important mechanism of tolerance induction in this system. However, chimeras with IL4-GFP donors and/or recipients also exhibited a modest accumulation of peripheral $CD8\alpha^{-}CD8\beta^{-}$ Jedi-TCRαβ cells (Fig. 3e and Supplementary Fig. 1a), suggesting that diversion to the innate-like lineage[1, 2, 3] may also contribute to the induction of tolerance in this setting. We also observed some peripheral accumulation of $CD8\alpha^{int}CD8\beta^{int}$ Jedi-TCRαβ cells in [IL4-GFP+Jedi-TCRαβ]→WT chimeras (Fig. 3e and Supplementary Fig. 1b). The latter phenotype was recently described in association with peripheral eviction of immature autoreactive thymocytes[4], possibly suggesting the contribution of this tolerance mechanism to the inactivation of autoreactive Jedi-TCRαβ cells. However, we did not observe the emergence of a peripheral $CD4^{int}CD8^{int}$ population (Fig. 3e) or upregulation of CD122 and Ly49 by autoreactive cells that has also been previously associated with this mechanism of CD8 T cell tolerance[4] (Supplementary Fig. 1c).

Accumulation of $CD4^{-}CD8^{-}$ TCR^{+} cells discussed above was reported for TCR-transgenic models with premature expression of a complete αβTCR at the DN stages of T cell development. In such settings, accumulation of TCR^{+} DN cells can be evident even in the absence of self antigens and is further enhanced in their presence[1, 5]. Indeed, thymic $CD8^{-}CD4^{-}$ Jedi-$TCR\alpha\beta^{+}$ cells were readily detectable in the absence of GFP (Fig. 3c). Analysis of T cell development in Jedi-TCRαβ mice at steady state demonstrated that the Jedi TCR was expressed

by a fraction of DN thymocytes, most of which had a $CD25^{-}CD44^{-}$ DN4-like surface phenotype (Supplementary Fig. 1d). However, unlike *bona fide* WT DN4 thymocytes, these cells upregulated CCR7 and initiated CD24 downregulation (Supplementary Fig. 1e), suggesting that they might represent precursors of peripheral DN cells rather than cells that will progress to the DP stage of T cell development. In line with the lineage diversion of some autoreactive cells with premature expression of the Jedi TCR, $CD8\alpha^{-}CD8\beta^{-}$ and $CD8\alpha^{+}CD8\beta^{-}$ Jedi-TCRαβ T cells were readily detectable among iIELs and were further increased in the presence of IL4-GFP (Supplementary Fig. 2).

We next sought to assess the induction of tolerance to the model inflammation-associated self antigen in a setting with physiological timing of TCR expression. To this end, we took advantage of the fact that mice that carry a pre-rearranged TCRβ chain of the Jedi TCR, but no Jedi TCRα chain (referred here and below as Jedi-TCRβ mice), contain a small distinct population of $H2\text{-}K^{d}$ $GFP_{200\text{-}208}$-specific thymocytes (Supplementary Fig. 1d). As development of this GFP-specific population relies on the recombination of the endogenous *Tcra* loci at the DP stage of T cell development, all GFP-specific cells were found exclusively in the DP and CD8SP compartments (Supplementary Fig. 1d). We next compared the fate of these GFP-specific cells in [WT+Jedi-TCRβ]→WT and [IL4-GFP+Jedi-TCRβ]→WT BM chimeras as well as in the corresponding chimera groups with Jedi-TCRαβ BM. This analysis revealed that in the presence of IL4-GFP, GFP-reactive Jedi-TCRβ T cells were virtually undetectable (decreased to background frequencies found in non-TCR transgenic controls) in the thymi, lymph nodes and even among iIELs of BM chimeras (Supplementary Fig. 2). These results suggest that physical elimination of the autoreactive cells in the thymus is the principal mechanism of tolerance induction in this

system with physiological timing of TCR expression. It is important to note that even in chimeras with a complete Jedi-TCRαβ, negative selection had contributed to the formation of tolerance. Indeed, [IL4-GFP+Jedi-TCRαβ]→WT BM chimeras exhibited a strong increase in the frequencies of DP thymocytes positive for active caspase 3 (Fig. 3d) as well as DP cells stained with viability dye (Fig. 3c). In the secondary lymphoid organs, this was also reflected by a strong decrease in Jedi-TCRαβ^{+} cells (Fig. 3e and Supplementary Fig. 2). Thus, while both negative selection and lineage diversion contributed to the induction of tolerance in a setting with premature TCR expression, no detectable contribution of the latter pathway was observed when timing of TCR expression was physiological.

Supplementary note 2. Analysis of *PLZF*$^{lu/lu}$ and *Tcrd*$^{-/-}$*Cd1d*$^{-/-}$*Mr1*$^{-/-}$ mice.

We sought to find a system in which innate-like T cells would be absent or nearly absent from the thymus. Unexpectedly, our analysis of IL4-, IFNγ- and IL17A-producing populations in the thymi of *Tcrd*$^{-/-}$*Cd1d*$^{-/-}$*Mr1*$^{-/-}$ mice lacking γδT cells, iNKT cells and MAIT cells did not reveal any decrease in the cytokine-producing populations (Supplementary Fig. 3a). These results mirrored the observations previously made in the secondary lymphoid organs of these mice[6] and suggested that in the absence of the largest non-conventional T cell populations their niches might be taken by other yet-to-be-characterized innate-like T cell or ILC subsets. Similar analysis of mice deficient for the transcription factor PLZF, a genetic model with broad, yet incomplete, deficiency in multiple innate-like T cell subsets[7, 8, 9, 10] likewise revealed normal frequencies of IFNγ- and IL17A-producing cells in the thymus (Supplementary Fig. 3b). IL4-producing thymocytes were decreased, but, consistent with previous observations[8], were still readily detectable in the PLZF-deficient thymi (Supplementary Fig. 3b). In line with that, and with the

redundancy between thymic innate-like T cells, eosinophils and TECs in induction of tolerance to IL4-GFP described above, repetitive "immunizations" of *PLZF*$^{lu/lu}$→*Rag2*$^{-/-}$ and WT→*Rag2*$^{-/-}$ BM chimeras with *in vitro* polarized T_H2 cells followed by an injection of CTV-labeled T_H2 cells for an *in vivo* killing assay failed to reveal rejection of this effector subset (Supplementary Fig. 4a). Finally, we used a custom bead-based antigen array to profile serum samples from WT and *PLZF*$^{lu/lu}$ mice for antibodies against cytokines. While antibodies against some cytokines were detectable in sera from MRL *Fas*$^{lpr/lpr}$ mice that were used as a positive control, no such response was reproducibly detected in PLZF-deficient animals (Supplementary Fig. 4b).

We conclude that none of the existing genetic models enables the complete elimination of thymic innate-like lymphocytes to study the effects of such defect on the induction of tolerance.

1. Egawa, T., Kreslavsky, T., Littman, D.R. & von Boehmer, H. Lineage Diversion of T Cell Receptor Transgenic Thymocytes Revealed by Lineage Fate Mapping. *PLoS ONE* **3**, e1512 (2008).

2. Bruno, L., Fehling, H.J. & von Boehmer, H. The alpha beta T cell receptor can replace the gamma delta receptor in the development of gamma delta lineage cells. *Immunity* **5**, 343-352 (1996).

3. Terrence, K., Pavlovich, C.P., Matechak, E.O. & Fowlkes, B.J. Premature expression of T cell receptor (TCR)alphabeta suppresses TCRgammadelta gene rearrangement but permits development of gammadelta lineage T cells. *J Exp Med* **192**, 537-548 (2000).

4. Badr, M.E., Zhang, Z., Tai, X. & Singer, A. CD8 T cell tolerance results from eviction of immature autoreactive cells from the thymus. *Science* **382**, 534-541 (2023).

5. Baldwin, T.A., Sandau, M.M., Jameson, S.C. & Hogquist, K.A. The timing of TCR alpha expression critically influences T cell development and selection. *J Exp Med* **202**, 111-121 (2005).

6. Ataide, M.A. *et al.* Lymphatic migration of unconventional T cells promotes site-specific immunity in distinct lymph nodes. *Immunity* **55**, 1813-1828.e1819 (2022).

7. Savage, A.K. *et al.* The Transcription Factor PLZF Directs the Effector Program of the NKT Cell Lineage. *Immunity* **29**, 391–403 (2008).

8. Kovalovsky, D. *et al.* The BTB-zinc finger transcriptional regulator PLZF controls the development of invariant natural killer T cell effector functions. *Nat Immunol* **9**, 1055 - 1064 (2008).

9. Alonzo, E.S. *et al.* Development of promyelocytic zinc finger and ThPOK-expressing innate gamma delta T cells is controlled by strength of TCR signaling and Id3. *J Immunol* **184**, 1268-1279 (2010).

10. Kreslavsky, T. *et al*. TCR-inducible PLZF transcription factor required for innate phenotype of a subset of gammadelta T cells with restricted TCR diversity. *Proc Natl Acad Sci U S A* **106**, 12453-12458 (2009).

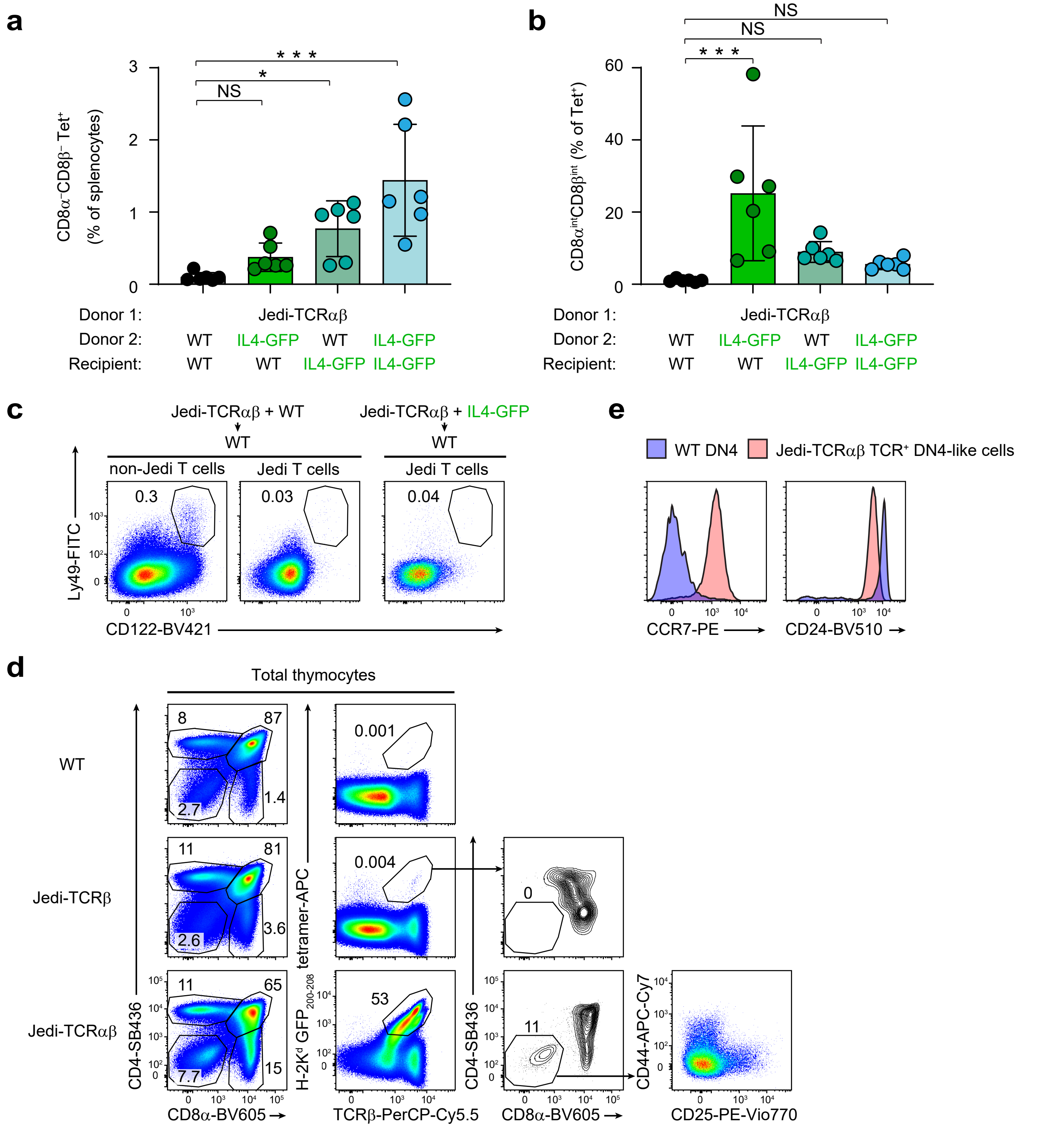

Supplementary Figure 1. Lineage diversion of Jedi-TCRαβ T cells. a. Frequency of CD8α⁻CD8β⁻ H2-K^{d} GFP$_{200\text{-}208}$ tetramer-binding cells of total splenocytes in spleens of BM chimeras for the experiment shown in Fig. 3e. **b**. Frequency of CD8α^{int}CD8β^{int} cells of H2-K^{d} GFP$_{200\text{-}208}$ tetramer-binding cells in spleens of BM chimeras for the experiment shown in Fig. 3e. n = 6 chimeras per group. Data are presented as mean ± SD with NS: non-significant (P > 0.05), *P < 0.05, ***P < 0.001. Data were analyzed by Kruskal Wallis test (a) or one-way ANOVA with Holm-Sidak's multiple comparisons test (b). **c**. Expression of CD122 and Ly49 by indicated populations in spleens of [WT+Jedi-TCRαβ]→WT and [IL4-GFP+Jedi-TCRαβ]→WT mixed BM chimeras. **d**. Flow cytometric characterization of the indicated thymic subsets in WT, Jedi-TCRβ and Jedi-TCRαβ mice. **e**. Expression of CCR7 and CD24 by WT DN4 cells (gated as TCR⁻CD4⁻CD8⁻CD25⁻CD44⁻, blue) and Jedi-TCRαβ⁺ DN4-like cells (gated as TCR⁺CD4⁻CD8⁻CD25⁻CD44⁻, red).

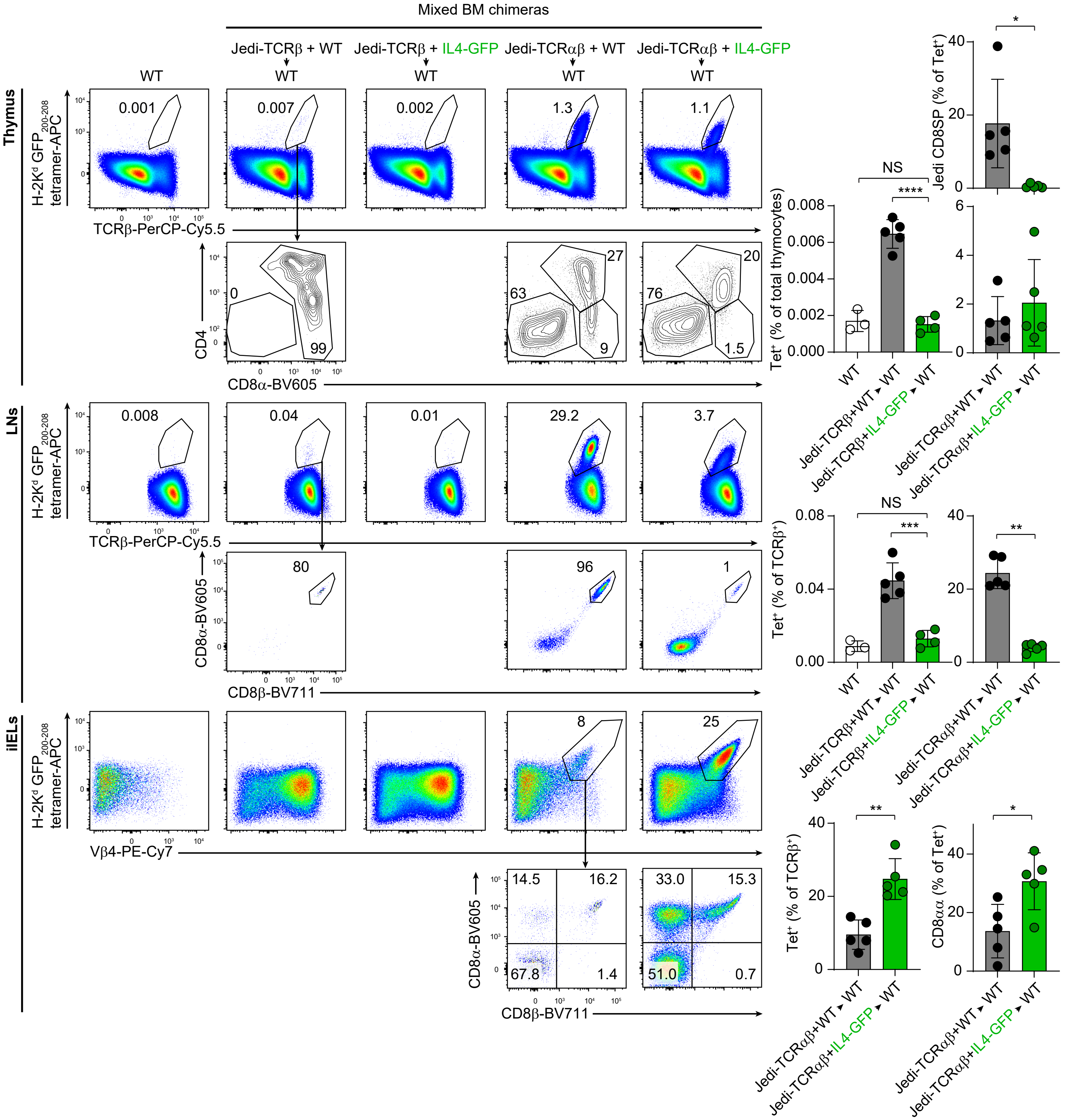

Supplementary Figure 2. Cell fates of GFP-specific Jedi-TCRβ and Jedi-TCRαβ T cells in mixed BM chimeras with or without IL4-GFP. [WT+Jedi-TCRβ]→WT, [IL4-GFP+Jedi-TCRβ]→WT, [WT+Jedi-TCRαβ]→WT and [IL4-GFP+Jedi-TCRαβ]→WT BM chimeras (all donors and recipients on BALB/c background) were established as described in Methods and analyzed 8 weeks after reconstitution. Thymi, LNs and iIELs were analyzed for presence and cell surface phenotype of H2-K^d GFP$_{200-208}$ tetramer-binding cells. Gating on total cells (thymus) or TCRβ$^+$ cells (LNs, IELs) was applied. One experiment with 4-5 BM chimeras (n = 3 mice for WT groups, n = 4 chimeras for thymus and LNs of [IL4-GFP+Jedi-TCRαβ]→WT group, n = 5 chimeras for the other groups). Data are presented as the mean ± SD with NS: non-significant ($P > 0.05$), *$P < 0.05$, **$P < 0.01$, and ****$P < 0.0001$. Data of thymus and LNs were analyzed by two-tailed Mann-Whitney test or one-way ANOVA with Holm-Sidak's multiple comparisons test. Data of iIELs were analyzed by two-tailed Student's t-test.

Supplementary Figure 2 (You *et al.*)

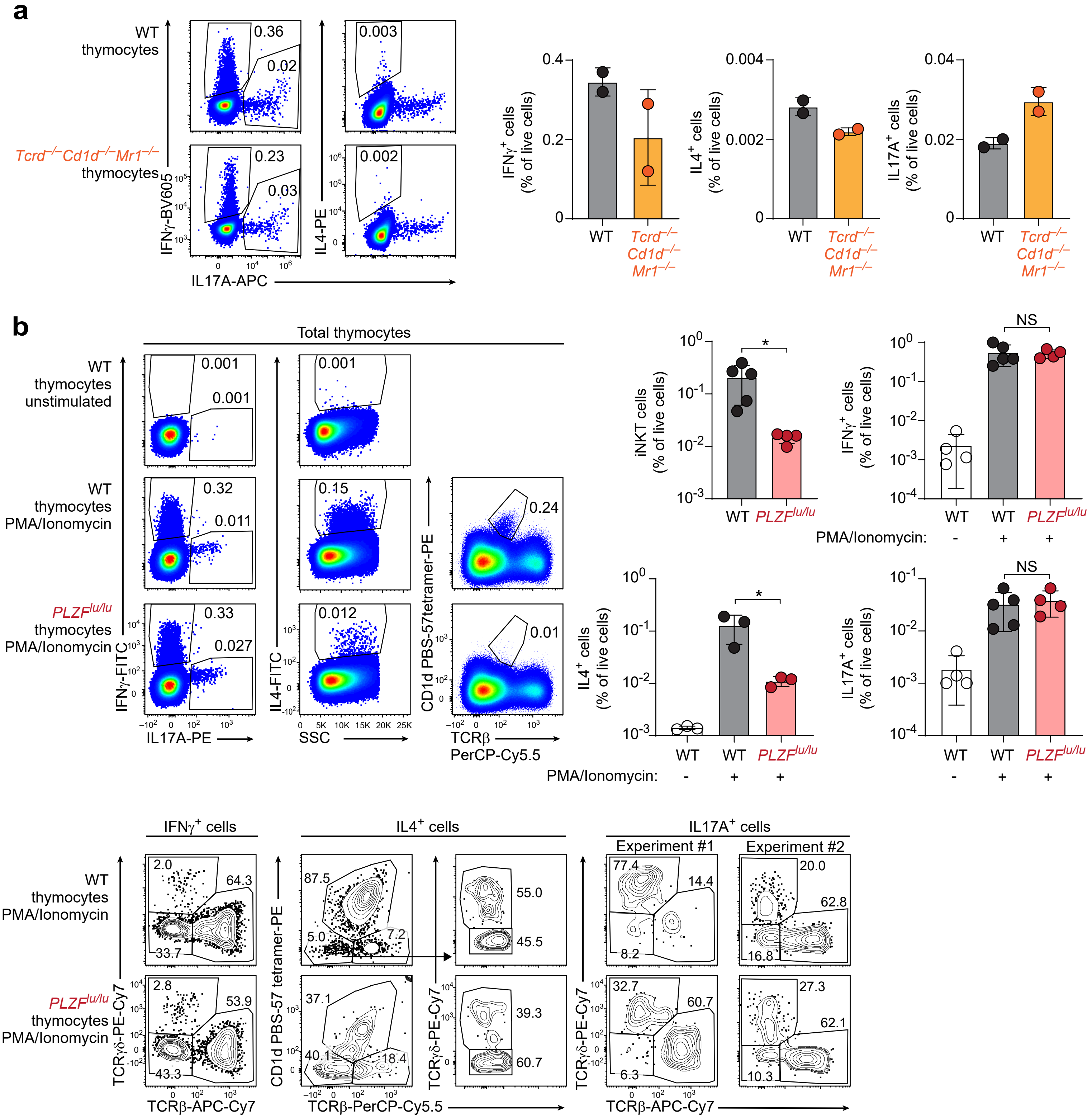

Supplementary Figure 3. Analysis of thymic effector populations in PLZF-deficient and *Tcrd*$^{-/-}$*Cd1d*$^{-/-}$*Mr1*$^{-/-}$ mice. a. Analysis of IFNγ, IL17A and IL4 production after PMA/Ionomycin stimulation of total thymocytes from WT and *Tcrd*$^{-/-}$*Cd1d*$^{-/-}$*Mr1*$^{-/-}$ mice. Flow cytometry plots show concatenated data from 2 mice. Representative results of three individual experiments (n = 2 mice per group). **b**. Analysis of CD1d-PBS-57 tetramer binding and IFNγ, IL17A and IL4 production after PMA/Ionomycin stimulation of total thymocytes from WT and *PLZF*$^{lu/lu}$ mice. Frequencies of iNKT cells, IFNγ^{+} and IL17A^{+} populations were pooled from several independent experiments (n = 4 mice for WT unstimulated group, n = 5 for WT stimulated group, n = 4 for *PLZF*$^{lu/lu}$ stimulated group). Results for IL4 production were obtained in a separate experiment (n = 3 per group). Bottom panels show the expression of TCR chains and CD1d-PBS-57 tetramer binding by the cytokine-producing populations. Examples from two experiments are shown for IL17A-producing cells to illustrate variability. Data are presented as the mean ± SD with NS: non-significant ($P > 0.05$) and *$P < 0.05$. Data were analyzed by two-tailed Student's t-test.

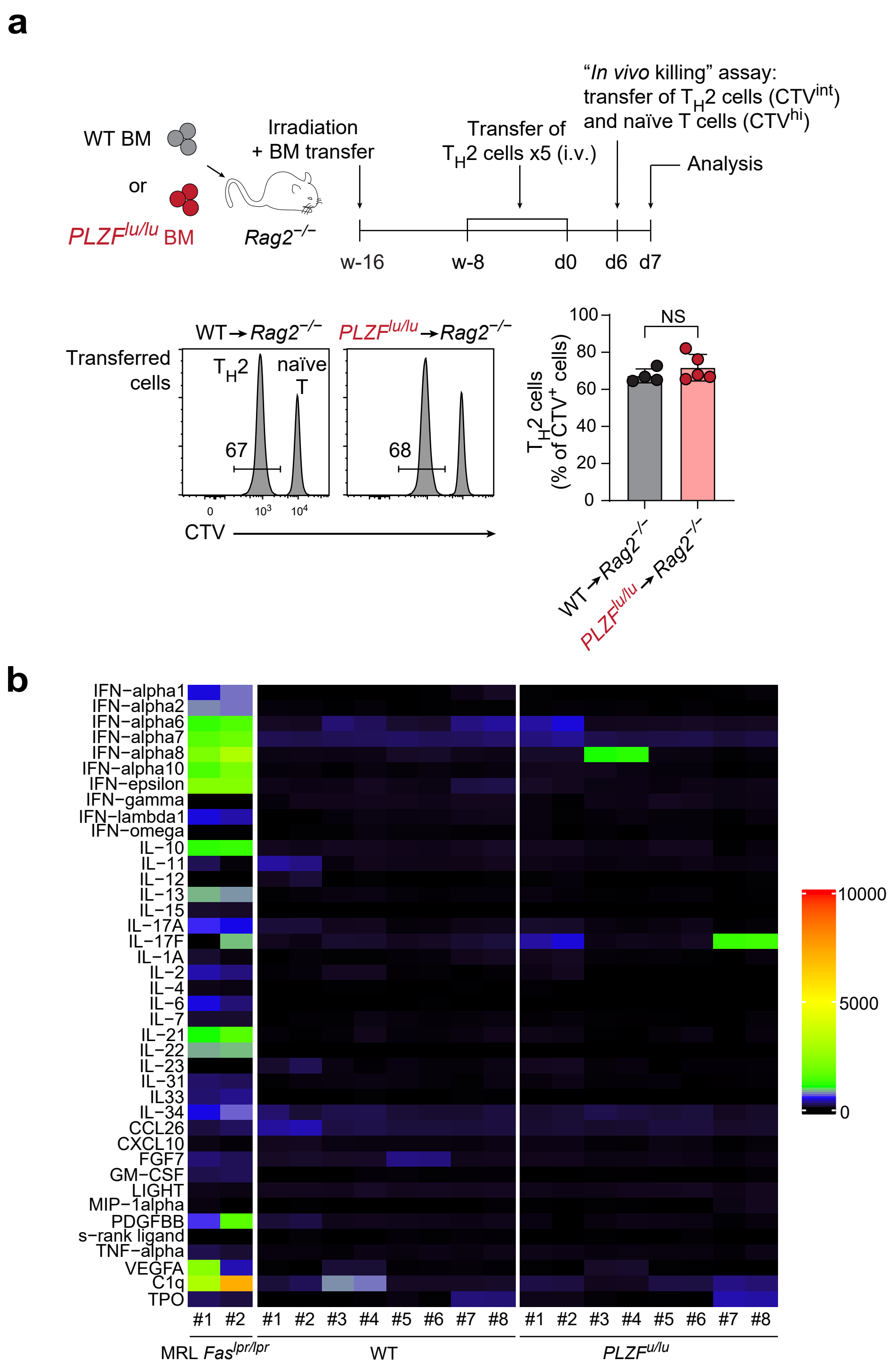

Supplementary Figure 4. Lack of autoimmune phenotype in PLZF-deficient mice. a. *Rag2*$^{-/-}$ mice (on C57BL/6 background) were lethally irradiated and reconstituted with syngeneic WT or *PLZF*$^{lu/lu}$ BM cells depleted of T- and NK-cells. Chimeras were injected i.v. with *in vitro* differentiated T$_H$2 cells five times between weeks 8 and 16 after BM reconstitution. "*In vivo* killing" assay was performed 6 days after the last injection by transferring *in vitro* differentiated T$_H$2 cells labeled with intermediate levels of CellTrace Violet (CTV) dye mixed with CTV^{hi} naïve T cells for normalization. The frequency of CTV^{int} T$_H$2 cells among total CTV^{+} cells was analyzed the next day. Representative results of three independent experiments (n = 4 chimeras for WT→*Rag2*$^{-/-}$, n = 5 for *PLZF*$^{lu/lu}$→*Rag2*$^{-/-}$). Data are presented as the mean ± SD with NS: non-significant ($P > 0.05$). Data were analyzed by two-tailed Student's t-test. **b**. A custom bead-based antigen array was used to profile serum samples from WT and PLZF-deficient mice for autoantibodies against cytokines. Sera from MRL *Fas*$^{lpr/lpr}$ mice were used as a positive control.
