# Supplementary material for: Direct presentation of inflammation-associated self-antigens by thymic innate-like T cells induces elimination of autoreactive CD8+ thymocytes
Source: Nat Immunol. 2024 Jul 11;25(8):1367–82. doi: 10.1038/s41590-024-01899-6 (PMC11291280; doi:10.1038/s41590-024-01899-6)
Supplement: Supplementary file 2 — Reporting Summary [file 41590_2024_1899_MOESM2_ESM.pdf]

nature portfolio

Corresponding author(s): Taras Kreslavsky

Last updated by author(s): Jun 14, 2024

Reporting Summary

Nature Portfolio wishes to improve the reproducibility of the work that we publish. This form provides structure for consistency and transparency in reporting. For further information on Nature Portfolio policies, see our Editorial Policies and the Editorial Policy Checklist.

Statistics

For all statistical analyses, confirm that the following items are present in the figure legend, table legend, main text, or Methods section.

n/a	Confirmed	
☐	☒	The exact sample size (n) for each experimental group/condition, given as a discrete number and unit of measurement
☐	☒	A statement on whether measurements were taken from distinct samples or whether the same sample was measured repeatedly
☐	☒	The statistical test(s) used AND whether they are one- or two-sided<br>*Only common tests should be described solely by name; describe more complex techniques in the Methods section.*
☐	☒	A description of all covariates tested
☐	☒	A description of any assumptions or corrections, such as tests of normality and adjustment for multiple comparisons
☐	☒	A full description of the statistical parameters including central tendency (e.g. means) or other basic estimates (e.g. regression coefficient) AND variation (e.g. standard deviation) or associated estimates of uncertainty (e.g. confidence intervals)
☐	☒	For null hypothesis testing, the test statistic (e.g. F, t, r) with confidence intervals, effect sizes, degrees of freedom and P value noted<br>*Give P values as exact values whenever suitable.*
☒	☐	For Bayesian analysis, information on the choice of priors and Markov chain Monte Carlo settings
☒	☐	For hierarchical and complex designs, identification of the appropriate level for tests and full reporting of outcomes
☒	☐	Estimates of effect sizes (e.g. Cohen's d, Pearson's r), indicating how they were calculated

Our web collection on statistics for biologists contains articles on many of the points above.

Software and code

Policy information about availability of computer code

Data collection	LSR Fortessa (BD Biosciences), FACSCanto™ II (BD Biosciences), Cytek Aurora (Cytek Biosciences), BD FACS Diva, Varioskan LUX Multimode Microplate Reader (ThermoFisher), LSM 800 system (Carl Zeiss), LSM880 microscope (Carl Zeiss), FlexMap3D instrument (Luminex Corp.).
Data analysis	FlowJo software v.10 (BD), Microsoft Office, Zen 2.3 Black Edition (Carl Zeiss), Imaris 10.1.0 (Bitplane), Scanpy 1.9.1, GraphPad Prism 9.

For manuscripts utilizing custom algorithms or software that are central to the research but not yet described in published literature, software must be made available to editors and reviewers. We strongly encourage code deposition in a community repository (e.g. GitHub). See the Nature Portfolio guidelines for submitting code & software for further information.

Data

Policy information about availability of data

All manuscripts must include a data availability statement. This statement should provide the following information, where applicable:

- Accession codes, unique identifiers, or web links for publicly available datasets
- A description of any restrictions on data availability
- For clinical datasets or third party data, please ensure that the statement adheres to our policy

Microarray datasets for Il4, Ifng, Gzmb expression by thymic cell subsets were obtained from ImmGen Consortium. Human and mouse thymus cell atlases were obtained from https://developmental.cellatlas.io/thymus-development.

Human research participants

Policy information about studies involving human research participants and Sex and Gender in Research.

Reporting on sex and gender	Not applicable
Population characteristics	Not applicable
Recruitment	Not applicable
Ethics oversight	Not applicable

Note that full information on the approval of the study protocol must also be provided in the manuscript.

Field-specific reporting

Please select the one below that is the best fit for your research. If you are not sure, read the appropriate sections before making your selection.

☒ Life sciences ☐ Behavioural & social sciences ☐ Ecological, evolutionary & environmental sciences

For a reference copy of the document with all sections, see nature.com/documents/nr-reporting-summary-flat.pdf

Life sciences study design

All studies must disclose on these points even when the disclosure is negative.

Sample size	No statistical test was used to pre-determine the sample size. For RTOC analyses, the sample size was dependent on the number of embryos and embryonic thymi that could be harvested, and the mating was set up based on prior experience to give sufficient number of embryos. For mouse phenotype analysis, the sample size was selected based on our previous experience and mouse availability.
Data exclusions	In Fig. 4b-d, 5c, 6a, Extended Data Fig. 3b, 3c, 6c, all RTOCs that were established with GFP-expressing cells but where no GFP+ cells were detectable during analysis, were excluded.<br>In Fig. 5d, all thymic lobes that were injected with IL4-GFP iNKT cells but where no GFP+ cells were detectable during analysis, were excluded.<br>In Fig. 6d-g, all DP thymocyte:iNKT cell interactions lasting for 2 frames or less were excluded from the analyses.
Replication	All experiments were repeated at least two times and gave similar results, with the exception of Caspase3 staining (Fig. 3d), WT->IL17A-GFP and IL17A-GFP->IL17A-GFP bone marrow chimera (Fig. 5b), RTOCs with IL4-GFP iNKT cells from different backgrounds (Fig. 6a), RTOCs with Actb-GFP CD4SP cells (Extended Data Fig. 3b) and IL4-GFP eosinophils (Extended Data Fig. 3c), and RTOCs with IL17A-GFP iNKT cells from different backgrounds (Extended Data Fig. 6b) which were analyzed in one experiment.
Randomization	After matching for age and sex, mice were randomly assigned to groups.
Blinding	Blinding was not feasible as most of the experiments were performed by one and the same person.

Reporting for specific materials, systems and methods

We require information from authors about some types of materials, experimental systems and methods used in many studies. Here, indicate whether each material, system or method listed is relevant to your study. If you are not sure if a list item applies to your research, read the appropriate section before selecting a response.

Materials & experimental systems

n/a	Involved in the study	
☐	☒	Antibodies
☐	☒	Eukaryotic cell lines
☒	☐	Palaeontology and archaeology
☐	☒	Animals and other organisms
☒	☐	Clinical data
☒	☐	Dual use research of concern

Methods

n/a	Involved in the study	
☒	☐	ChIP-seq
☐	☒	Flow cytometry
☒	☐	MRI-based neuroimaging

Antibodies

Antibodies used

Antibody/company/Dilution/Cat
Anti-mouse CD4, SB436 (RM4-8), Invitrogen (eBio)1:200 cat: 62-0042-82
Anti-mouse CD4, VioGreen (GK1.5), Miltenyi 1:50 cat: 130-123-899
Anti-mouse CD4, BV650 (GK1.5), Biolegend 1:200 cat: 100469
Anti-mouse CD4, APC (GK1.5), Biolegend 1:200 cat:100412
Anti-mouse CD4, APCCY7 (GK1.5), Biolegend 1:200 cat:100413
Anti-mouse CD8α PECY7 (53-6.7), Biolegend 1:200 cat: 100722
Anti-mouse CD8α BV605 (53-6.7), Biolegend 1:200 cat: 100744
Anti-mouse CD8β APC (YT5156.7.7), Biolegend 1:200 cat: 126614
Anti-mouse CD8β APCCY7 (YT5156.7.7), Biolegend 1:200 cat: 126620
Anti-mouse CD8β BV711 (YT5156.7.7), Biolegend 1:200 cat: 126633
Anti-mouse PD-1 APC (REA802), Miltenyi 1:50 cat: 130-111-801
Anti-mouse PD-1 PE (29F.1A12), Biolegend 1:200 cat: 135205
Anti-mouse CD24 APC (M1/69), Biolegend 1:200 cat: 101813
Anti-mouse CD24 BV510 (M1/69), Biolegend 1:200 cat: 101831
Anti-mouse CD44 Pacific Blue (IM7), Biolegend 1:400 cat: 103019
Anti-mouse CD44 APCCY7 (IM7), Biolegend 1:400 cat: 103027
Anti-mouse CD19 APC (6D5), Biolegend 1:200 cat: 115512
Anti-mouse CD19 BV786 (1D3), BD 1:200 cat: 563333
Anti-mouse CD11c APC (N418), Biolegend 1:200 cat: 117310
Anti-mouse CD45.1 PerCP cy5.5 (A20), Biolegend 1:200 cat: 110726
Anti-mouse CD45.1 BV421 (A20), Biolegend 1:200 cat: 110732
Anti-mouse CD45.1 BV605 (A20), Biolegend 1:200 cat: 110738
Anti-mouse CD45.1 FITC (A20), Biolegend 1:200 cat: 110705
Anti-mouse CD45.2 PECY7 (104), Biolegend 1:200 cat: 109830
Anti-mouse CD45.2 APCCY7 (104), Biolegend 1:200 cat: 109823
Anti-mouse TCRβ Pacific Blue (H57-597), Biolegend 1:200 cat: 109226
Anti-mouse TCRβ Percpcy5.5 (H57-597), Biolegend 1:200 cat: 109228
Anti-mouse TCRγδ PE-Cy7 (GL3), Biolegend 1:200 cat: 118124
Anti-mouse H-2Kb APC (AF 6-885), BioLegend 1:200 cat: 116517
Anti-mouse H-2Kd PE (SF1-1.1), BioLegend 1:200 cat: 116607
Anti-mouse cKit BV421 (2B8), Biolegend 1:200 cat:105828
Anti-mouse Ter119 APC (TER-119), Biolegend 1:200 cat: 116212
Anti-mouse NK1.1 APC (S17016D), Biolegend 1:200 cat: 156506
Anti-mouse SiglecF PE (E50-2440), BD Bioscience 1:200 cat: E50-2440
Anti-mouse MHCII BV421 (M5/114.15), Biolegend 1:200 cat: 107632
Anti-mouse MHCII PE-Vio770 (REA813), Miltenyi 1:200 130-112-389
Anti-mouse Ly51 PEcy7 (6C3), Biolegend 1:200 cat: 108313
Anti-mouse EPCAM APC (G8.8), Biolegend 1:200 cat: 118214
Anti-mouse TCRVα2 Pacific Blue (B20.1), Biolegend 1:200 cat: 127815
Anti-mouse TCRV β5.1, 5.2 APC (MR9-4), Biolegend 1:200 cat: 139505
Anti-mouse TCRVβ4 (REA729), Miltenyi 1:50 cat: 130-111-101
Anti-mouse Thy1.2 AF700 (30-H12), Biolegend 1:200 cat: 105319
Anti-mouse Thy1.1 Pacific Blue (OX7), Biolegend 1:400 cat: 202521
Anti-mouse Thy1.1 PE (OX7), Biolegend 1:200 cat: 202523
Anti-mouse Thy1.1 VioGreen (OX7), Miltenyi 1:50 cat: 130-112-879
Anti-mouse Ly49 FITC (14B11), Biolegend 1:200 cat:108205
Anti-mouse CD122 BV421 (TM-β1), BD 1:50 cat: 562960
Anti-mouse CCR7 PE (4B12), Miltenyi 1:100 cat: 130-126-035
Anti-mouse CD25 PEVio770 (REA568), Miltenyi 1:50 130-123-893
Anti-mouse CD80 PE (16-10A1), BD Biosciences 1:400 cat:553769
Anti-mouse CD86 PE (GL1), BD Biosciences 1:400 cat:553692
Anti-mouse CD80 PECy7 (16-10A1), Biolegend 1:200 cat:104733
Anti-mouse CD86 PECy7 (PO3.3), Miltenyi 1:50 cat: 130-116-518
PE Armenian Hamster IgG Isotype Ctrl (HTK888), Biolegend 1:400 cat:400907
PE/Cyanine7 Rat IgG2b, κ Isotype Ctrl (RTK4530), Biolegend 1:200 cat:400617
Anti-mouse IFNγ (REA638), Miltenyi 1:100 cat: 130-117-668
Anti-mouse IL4 FITC (11B11), Biolegend 1:200 cat: 504109
Anti-mouse IL17A PE (TC11-18H10) ,Miltenyi, 1:50 cat: 130-103-015
Anti-mouse caspase3 unconjugated (5A1E), Cell Signaling Technology 1:6400 cat: 9664S
Anti-rabbit IgG (H+L), F(ab')2 fragments PE,Cell Signaling Technology 1:1000 cat: 79408S
Streptavidin-APC ,BioLegend 1:500 cat: 405207
Biotinylated UEA1, Vector Laboratories 1:200. cat: B-1065

Validation

All antibodies are commercially available and validated by the vendors. Validation data are available on the vendors' websites.

Eukaryotic cell lines

Policy information about cell lines and Sex and Gender in Research

Cell line source(s)	16.2c11 reporter cell line is a kind gift from Jan Kisielow. HEK293T cells were obtained from Research Institute of Molecular Pathology (IMP), Vienna.
Authentication	Cell lines were not authenticated.
Mycoplasma contamination	Cell lines were tested negative for mycoplasma.
Commonly misidentified lines (See ICLAC register)	No commonly misidentified lines were used.

Animals and other research organisms

Policy information about studies involving animals; ARRIVE guidelines recommended for reporting animal research, and Sex and Gender in Research

Laboratory animals	IL4-GFP, IL17A-GFP and Ifng–/– mice were purchased from the Jackson Laboratory. Rag2–/– mice were purchased from Janvier Labs. PLZFlu/lu, OT-I, Actb-GFP, Jedi-TCRαβ and Tcrd–/–Cd1d–/–Mr1–/– mice were described previously. WT C57BL/6J and BALB/c mice were obtained from Janvier Labs or bred in house. Jedi-TCRβ only mice were generated from Jedi-TCRαβ mice by crossing with WT BALB/c mice and were further backcrossed to BALB/c background for more than 10 generations. WT C57BL/6J x BALB/c F1 mice (referred to as CB6F1), IL4-GFP CB6F1, IL17A-GFP CB6F1, Actb-GFP CB6F1 and Jedi-TCRβ CB6F1 mice were generated by crossing mice on C57BL/6J and BALB/c backgrounds. For MHC mismatch experiments, IL4-GFP CB6F1 mice were crossed with WT C57BL/6J mice to generate IL4-GFP H2b/d and IL4-GFP H2b/b mice. Tcrd–/–Cd1d–/–Mr1–/– mice were bred and maintained at the Würzburg Institute for Systems Immunology. All other mice were bred and maintained at the Comparative Medicine Biomedicum facility of Karolinska Institutet (Stockholm, Sweden). All mice were housed in SPF condition at 50% humidity, 22°C, with 6pm to 6am nocturnal dark light circle.
Wild animals	No wild animals were used in this study.
Reporting on sex	Both male and female mice were analyzed.
Field-collected samples	This study did not include any field-collected samples.
Ethics oversight	All mouse experiments were carried out according to valid project licenses, which were approved and regularly controlled by the Swedish Veterinary Authority.

Note that full information on the approval of the study protocol must also be provided in the manuscript.

Flow Cytometry

Plots

Confirm that:

☒ The axis labels state the marker and fluorochrome used (e.g. CD4-FITC).

☒ The axis scales are clearly visible. Include numbers along axes only for bottom left plot of group (a 'group' is an analysis of identical markers).

☒ All plots are contour plots with outliers or pseudocolor plots.

☒ A numerical value for number of cells or percentage (with statistics) is provided.

Methodology

Sample preparation	Mouse organs were freshly harvested, and single cell suspensions were obtained by mincing through 70 µm cell strainers. Isolation of iIEL was performed as described previously.
Instrument	LSR Fortessa Flow Cytometer, FACS Aria III (BD Biosciences), or Cytek Aurora (Cytek Biosciences)
Software	Data collection: FACS Diva (v.8)<br>Data analysis: FlowJo (v.10)
Cell population abundance	The abundance of the post-sort fraction was determined by reanalysis.
Gating strategy	Live gate (FSC/SSC) included all live cells: from small lymphocytes to larger monocytes and SSC-hi granulocytes. For the most part, erythrocytes were gated out. Detailed gating strategies are described in the figures and figure legends.

☒ Tick this box to confirm that a figure exemplifying the gating strategy is provided in the Supplementary Information.
